# “It’s all about asking from those who have walked the path”: Patient and stakeholder perspectives on how peers may shift substance use stigma in HIV care in South Africa

**DOI:** 10.1186/s13722-022-00330-5

**Published:** 2022-09-21

**Authors:** Jessica F. Magidson, Alexandra L. Rose, Kristen S. Regenauer, Carrie Brooke-Sumner, Morgan S. Anvari, Helen E. Jack, Kim Johnson, Jennifer M. Belus, John Joska, Ingrid V. Bassett, Goodman Sibeko, Bronwyn Myers

**Affiliations:** 1 Department of Psychology, University of Maryland, 4094 Campus Drive, College Park, Maryland, MD USA; 2 Alcohol, Tobacco and Other Drug Research Unit, South African Medical Research Council, Parow, South Africa; 3 Division of General Internal Medicine, Department of Medicine, University of Washington, Seattle, WA USA; 4 Department of Clinical Research, University Hospital Basel, Basel, Switzerland; 5 HIV Mental Health Research Unit, Department of Psychiatry and Mental Health, University of Cape Town, Cape Town, South Africa; 6 Division of Infectious Diseases, Medical Practice Evaluation Center, Massachusetts General Hospital/Harvard Medical School, Boston, MA USA; 7 Curtin enAble Institute, Faculty of Health Sciences, Curtin University, Building 408, GPO Box U1987, Perth, WA 6845 Australia; 8 University of Basel, Basel, Switzerland

**Keywords:** Peer, Substance use stigma, Substance use treatment, HIV stigma, Global mental health, Implementation science

## Abstract

**Background:**

South Africa has the highest number of people with HIV (PWH) globally and a significant burden of co-occurring substance use disorder (SUD). Health care worker (HCW) stigma towards SUD is a key barrier to HIV care engagement among PWH with SUD. Support from peers—individuals with lived experience of SUD—may be a promising solution for addressing SUD stigma, while also improving engagement in HIV care. We evaluated the perceived acceptability of integrating a peer role into community-based HIV care teams as a strategy to address SUD stigma at multiple levels and improve patient engagement in HIV care.

**Methods:**

Patients and stakeholders (*N* = 40) were recruited from publicly-funded HIV and SUD organizations in Cape Town, South Africa. We conducted a quantitative assessment of stigma among stakeholders using an adapted Social Distance Scale (SDS) and patient perceptions of working with a peer, as well as semi-structured interviews focused on experiences of SUD stigma, acceptability of a peer model integrated into community-based HIV care, and potential peer roles.

**Results:**

On the SDS, 75% of stakeholders had high stigma towards a patient with SUD, yet 90% had low stigma when in recovery for at least 2 years. All patients endorsed feeling comfortable talking to someone in recovery and wanting them on their HIV care team. Three main themes emerged from the qualitative data: (1) patient-reported experiences of enacted SUD and HIV stigmas were common and impacted HIV care engagement; (2) both patients and stakeholders considered a peer model highly acceptable for integration into HIV care to support engagement and address SUD stigma; and (3) patients and stakeholders identified both individual-level and systems-level roles for peers, how peers could work alongside other providers to improve patient care, and key characteristics that peers would need to be successful in these roles.

**Conclusions:**

Findings from this formative work point to the promise of a peer model for reducing SUD stigma among patients and HCWs within community-based HIV care teams in SA.

**Supplementary Information:**

The online version contains supplementary material available at 10.1186/s13722-022-00330-5.

## Introduction

South Africa (SA) has the highest global burden of HIV, with over 7.8 million people with HIV (PWH) living in SA [1]. Untreated substance use disorder (SUD) is prevalent in SA and a significant barrier to sustained engagement in HIV care and antiretroviral therapy (ART) adherence [2, 3]. However, few interventions to support engagement in HIV care among people with SUD have been developed, tested, and taken to scale.

Stigma among health care workers (HCWs) surrounding SUD is a key barrier to improving HIV care outcomes among PWH with SUD. HCW stigma toward SUD prevents the delivery of SUD services and contributes to poor engagement in HIV care among PWH with SUD [4, 5]. Prior work by our team [4, 6] and others [5] found that HCW stigma towards PWH and SUD results in inferior HIV care delivery, including less time spent with patients, lower likelihood of delivering evidence-based and patient-centered care, and reluctance to screen for SUD or provide brief interventions [5, 7]. In our prior work [4, 6, 8], PWH with SUD described being scolded or judged by HCWs and stopping HIV care prematurely due to anticipated stigma. When stigma is internalized—i.e., individuals endorse negative beliefs towards themselves [9–11]—internalized SUD stigma is a barrier to HIV care engagement for patients [12–14]. Further, the intersection of SUD and HIV stigmas—i.e., when both SUD and HIV stigmas co-occur and converge upon each other [15, 16]—can exacerbate poor HIV and SUD treatment outcomes [15, 17, 18]. Yet, scarce research has explored the experiences of intersecting SUD and HIV stigmas in HIV care. Strategies to address both HIV and SUD stigmas among HCWs and patients are needed to improve engagement in HIV care among people with SUD.

Peers, individuals with lived experiences in SUD recovery, may be a promising solution for addressing SUD stigma among HCWs and patients, while also improving engagement in HIV care for PWH with SUD [19]. By bringing their shared experience and identity to interactions with patients (and other HCWs), peers may offer patients destigmatizing care and support integration of SUD services into HIV care. Although strategies for reducing SUD stigma have rarely been evaluated in low- and middle-income countries (LMICs), the limited evidence available on mental health stigma suggests that contact with peers is an effective way of reducing mental health-related stigma among HCWs [20]. Further, through shared lived experience, peers may reduce internalized stigma among patients and HCW stigma [20]. Despite their promise, limited research has tested whether peers can shift SUD stigma among patients and HCWs to improve HIV care outcomes among people with SUD, especially in LMICs with substantial HIV burden.

In higher income settings, such as the US, formal models of peer-delivered services for patients with SUD have scaled rapidly (i.e., “peer recovery coaches” or “peer recovery specialists”) [21–23]. Peer recovery coaches or specialists typically assist with health services navigation, advocacy, and outreach, while also supporting motivation and ongoing recovery through shared lived experience with SUD [24, 25]. Formal peer models are often differentiated from more informal peer services based on: (1) the formal peer role typically being a paid position; (2) having a unidirectional emphasis (i.e., focused on support to others vs. mutual support, such as 12 step, where the focus is bidirectional); and/or 3) certification that involves structured training and supervision [21, 26]. These certification programs are typically short (days to weeks) and cover the peer recovery coach role, ethics and boundaries, motivational interviewing strategies, including stages of change, and recovery wellness planning, with substantial peer expertise coming directly from their own lived experience. Although formal peer certification programs are rapidly expanding in the US and other high-income settings, a recent systematic review conducted by our team [19] found that more formal peer training programs for SUD are lacking in LMICs, where the focus has been on less structured interventions and/or peer roles. Our team’s prior review [19] identified examples of peer-delivered services for SUD in LMICs, with results pointing to promising effects on HIV prevention outcomes, but less of a focus on SUD outcomes, and few examples of structured, formalized peer roles integrated into health care teams. There have been few efforts to integrate more formal peer-delivered services for SUD within HIV care in LMICs, despite the potential for improving HIV care engagement. Mixed methods implementation science research is needed to develop strategies to promote integration of peers into health care teams [24]. Further, we are not aware of any studies that have explored the integration of peers focused on SUD into HIV care as a strategy for reducing SUD stigmas at multiple levels (i.e., HCW- and patient-levels) and improving HIV care engagement among people with SUD.

**Fig. 1:**
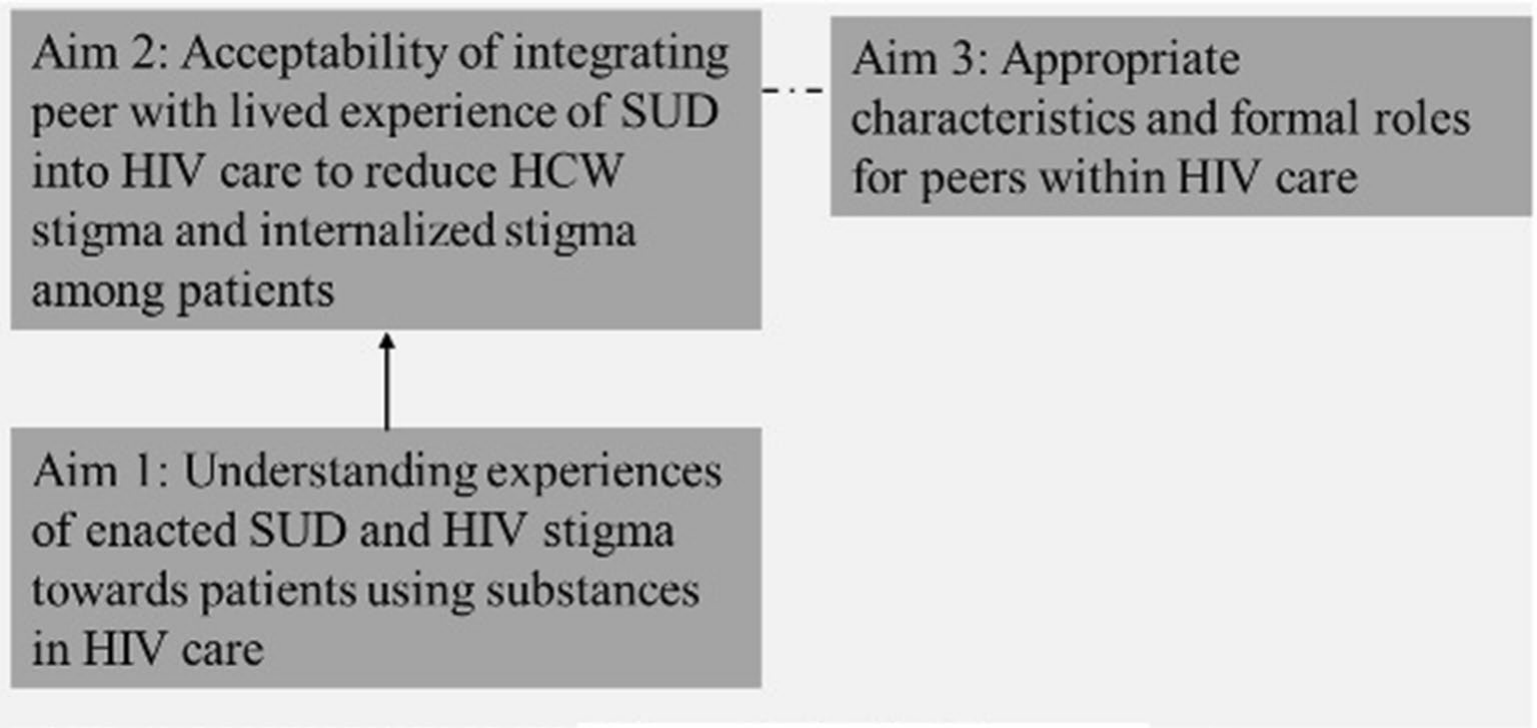
Conceptualization of the links between study aims

As a first step towards developing a more structured peer model that is acceptable to patients and providers and feasible for use in South African community HIV services, this study aimed to explore HCW and patient perspectives of: (1) experiences of both SUD and HIV stigmas in community-based HIV care in SA; (2) the acceptability of integrating peers with lived experience of SUD to address these stigmatizing experiences in community-based HIV care and enhance HIV care engagement for people with SUD; and (3) appropriate characteristics and responsibilities for peers to be successful in this role (see Fig. 1 for a conceptualization of the links between study aims).

## Materials and methods

Quantitative assessments and semi-structured, in-depth interviews (*N* = 40) were conducted between February and June 2021 with patients (*n* = 15) and stakeholders (*n* = 25) from HIV and SUD care services in low-income areas outside Cape Town, SA with high HIV and SUD burden. Purposeful sampling was used to recruit participants with knowledge relevant to the study aims, including typical patient cases and key stakeholders, detailed below. The intention was not to recruit a sample that would generalize to the population of all stakeholders but rather to generate a diversity of perspectives relevant to implementing a peer model [27].

### Participants

Stakeholders were identified based on referral from the Western Cape Provincial Department of Health and Wellness (WCDoH), which oversees relevant health care services in the region. Stakeholders were included if they held any of the following positions: a) directly involved in community-based HIV care services; b) a facility-based HIV provider who interacted with community-based teams; c) involved in planning for community-based HIV service delivery; or d) involved in SUD treatment co-located alongside public HIV clinics. All stakeholder participants worked in state or non-profit organizations receiving public funding, were ≥ 18 years old, and able to complete informed consent and interviews in isiXhosa or English. Study staff then contacted key individuals who would be eligible for an interview to complete informed consent.

Patients were included if they self-reported: (1) being ≥ 18 years old; (2) living with HIV; (3) struggling with HIV care engagement (e.g., missed appointments or challenges with ART adherence); (4) active substance use (i.e., ≥ 2 on the AUDIT-C [28]; or using ≥ 1 illicit drug in the past 3 months); and [5] ability to complete informed consent and interviews in isiXhosa or English. Typical patient cases were purposefully identified from stakeholder referrals. Interested patients provided consent before being screened; eligible participants were then consented for the interview.

### Procedures

All assessments and interviews were conducted by research assistants (RAs) trained in qualitative interviewing. Interviews were conducted in English or isiXhosa based on participant preference. Before beginning the interview, RAs obtained voluntary informed consent, collected demographic information, and quantitatively assessed participants’ perceptions of interacting with people with HIV and SUD. Specifically, stakeholders’ perceptions were assessed using an adapted Social Distance Scale (SDS) [29, 30]. The modified SDS included a vignette about Andile, a patient living with HIV and SUD, specifically tik (methamphetamine), who was struggling to take care of himself (see Supplementary Material for description of Andile vignette). Stakeholder participants answered six modified SDS questions about interacting with Andile personally, and three additional questions about interacting with Andile if he was in SUD recovery for at least two years. There were four response options for each question (i.e., definitely not, unlikely, likely, or definitely—reflecting either a desire or lack of desire for proximity to Andile). Patient participants were given a similar vignette about Andile, although in this version he received SUD treatment and was successfully adhering to his HIV medication. Patients then answered four questions pertaining to their preferences for involving Andile as part of their care.

RAs then followed semi-structured interview guides to ask about experiences of stigma surrounding HIV and alcohol/other drug use; acceptability of working with peers to address SUD stigma and support engagement in HIV care; and attitudes towards working with a peer. Separate guides were developed for stakeholders and patients in both isiXhosa and English. Both interview guides were informed by the Link and Phelan stigma framework, the Situated Information Motivation Behavioral Skills Model of Care Initiation and Maintenance, and the Consolidated Framework for Implementation Research [31–33]. A brief description of peer recovery models was provided to patients and stakeholders, specifying that peers are trained individuals who share common identities with patients (i.e., SUD) who would be integrated into the care team to help improve health outcomes for people with HIV and SUD. Interviews were audio-recorded and lasted approximately 45 min. Participants were compensated 150ZAR in grocery vouchers. All procedures were approved by the Human Research Ethics Committee at the South African Medical Research Council with an IRB Authorization Agreement with the University of Maryland.

### Data analysis

For the quantitative measure of social distance, descriptive statistics were used. Specifically, we scored the SDS by categorizing stakeholder participants into three categories (*high*, *moderate*, or *low* stigma), based on previously used scoring methods [34, 35]. *High* was scored as ≥ 2 “undesirable” responses (out of 6 total), *moderate* was scored as 1 “undesirable” response, and low was 0 (see Table 2).

For the semi-structured interviews, the interviews conducted in isiXhosa were first translated into English and transcribed verbatim by a bilingual translator. All transcripts were double checked for quality assurance. Memos were written to summarize cross-cutting themes following the aims of our study [36]. Guided by thematic analysis [37] and the theoretical frameworks informing the interview guide [31–33], the coding team—comprised of an SA-based faculty member, a US-based doctoral student, and a US-based research assistant—identified three key themes that mapped onto study aims: (1) experiences of SUD and HIV stigmas and their impact on HIV care engagement; (2) acceptability of incorporating peers in SUD recovery into HIV care; and (3) peer roles and characteristics. Initial coding was deductive based on primary study aims to examine experiences of SUD stigma and how peers may play a role in shifting stigma at multiple levels within community-based HIV care; yet, inductive approaches were also used to allow new themes to emerge from the data [38]. Data analysis was iterative and interviews were conducted and analyzed until theoretical saturation was reached. A codebook was created based on an initial three interviews with input from the local team and modified as new concepts arose [39]. Two independent coders coded each interview using NVivo. Coding comparisons were run in NVivo. Coding discrepancies were reviewed and resolved by discussion. A multidisciplinary team reviewed all codes and memos weekly, and through discussion identified cross-cutting themes following axial coding procedures.

## Results

Stakeholder participants were mostly mixed race (48%) or Black African (40%), mostly female (68%), and had been in their current job for an average of 4 years (*SD* = 1.00). Occupations included directors/managers/supervisors (*n* = 12), SUD counselors (*n* = 3), community health workers/field workers (*n* = 5), nurses (*n* = 2), and HIV counsellors (*n* = 3). Patients were 100% Black African, mostly female (73%), and had a mean AUDIT-C score of 7 (*SD* = 3.14). Only one patient reported prior SUD treatment. See Table 1 for more detail.

**Table 1:** Patient and stakeholder characteristics

Characteristic	n (%)
**Patients** **(*n* = 15)**	
Black African	15 (100)
Female	11 (73)
Age (mean [*SD*])	40.1 (8)
AUDIT-C score (mean [*SD*])	7 (3.14)
**Stakeholders** **(*n* = 25)**	
Black African	10 (40)
Mixed race	12 (48)
Female	17 (68)
Age (mean [*SD*])	41.2 (8.8)
Years in role (mean [*SD*])	4 (1)
Director/manager/supervisor	12 (48)
SUD counselor	3 (12)
CHW/field worker	5 (20)
Nurse	2 (8)
HIV counselor	3 (12)

On the SDS, most stakeholders scored high in social distance for questions about interacting with Andile when he was not in recovery (75%), yet low in social distance when he was in recovery for at least 2 years (90%). Stakeholders reported high interest in getting training to help a person like Andile (*M* = 9.45/10, *SD* = 1.54), most (70%) reported having family members with substance use or mental health concerns, and 40% reported having received SUD/mental health training in the past. See Table 2. All patients endorsed feeling comfortable talking to someone like Andile and wanting someone like him on their HIV care team. See Table 3. Tables 2 and 3 present additional quantitative results for stakeholders and patients.

**Table 2:** Stakeholder social distance scale (SDS) results

	Undesirable			Desirable		
	Definitely not	Unlikely		Likely		Definitely
Would you have a conversation with Andile?	1 (4%)	1 (4%)		1 (4%)		22 (88%)
Would you work on the same job as Andile?	5 (20%)	5 (20%)		3 (12%)		12 (48%)
Would you make friends with Andile?**	3 (12%)	6 (24%)		6 (24%)		9 (36%)
Would you share a room or a house with Andile?**	6 (24%)	11 (44%)		5 (20%)		2 (8%)
Would you date or marry someone like Andile?	15 (60%)	5 (20%)		5 (20%)		0

	Definitely	Likely		Unlikely		Definitely not
Would you be ashamed if people knew someone like Andile was in your family? [reverse coded]	1 (4%)	0		5 (20%)		19 (76%)

**SDS items in recovery**						
**If someone like Andile had now been in recovery from drug and alcohol use for 2 years:**						
	Undesirable			Desirable		
	Definitely not	Unlikely		Likely		Definitely
Would you want to work professionally with them as part of your team?	1 (4%)	0		2 (8%)		22 (88%)
Would you feel comfortable with them on your team?	0	0		4 (16%)		21 (84%)
Would you feel comfortable socializing with them?	0	1 (4%)		7 (28%)		17 (68%)

SDS Results Summary Based on Recovery Status						
	High social distance (≥ 2 Undesirable)		Moderate social distance (1 Undesirable)		Low social distance (0 Undesirable)	
Not in recovery	18 (72%)		6 (24%)		1 (4%)	
In recovery	0		2 (8%)		23 (92%)	

Other relevant questions						
			*M* (SD) or *n* (%)		Range	
Interested in getting training to help someone like Andile with drug problems** (1 not at all interested to 10 very interested*)*			9.54 (1.41)		5–10	
Has someone in family with mental health/substance use problems			18 (72%)		–	
Has received substance use or other mental health training			11 (44%)		–	

^*^The SDS is presented as the frequency of responses for each question, with four options ranging from definitely not to definitely, categorizing proximity as either “desirable” or “undesirable”

*High* SDS =  ≥ 2 undesirable responses (out of 6 total), *moderate* = 1 undesirable response, and *low* = 0

**1 participant declined to answer question

**Table 3:** Patient social distance scale (SDS) results

	Definitely not	Unlikely		Likely		Definitely
Would you feel comfortable talking with Andile about your own alcohol or other drug use?	0	0		3 (20%)		12 (80%)
Would you feel comfortable talking with Andile about any challenges with your HIV treatment?	0	0		1 (7%)		14 (93%)
	**Less interested**	**More interested**		**Unsure**		**Does not affect**
Does Andile’s history of drug use and recovery affect whether you want him as part of your HIV care team?	0	14 (93%)		0		1 (7%)
Does Andile’s HIV status affect whether you want him as part of your HIV care team?	0	14 (93%)		0		1 (7%)
			***M*** **(*SD*)** **or** ***n*** **(%)**		**Range**	
Interested in speaking to someone like Andile about alcohol or other drug problems (1 not at all interested to 10 very interested)			9.53 (1.36)		5–10	
Someone in their family like Andile			3 (20%)		–	
Someone among their friends like Andile			5 (33%)		–	
Number of years would want Andile to be in recovery before joining HIV care team			2.14 (1.45)		6mo–7 years	
Ever spoken to a peer, or someone like Andile, as part of HIV care?^1^			5 (33%)		–	
Ever spoken to a peer, or someone like Andile, as part of substance use treatment?^2^			4 (27%)		–	

^1^Counsellor at the clinic in risk of treatment failure group (*n* = 2); Cannot remember (*n* = 1); Peers and therapists at SU treatment (*n* = 1); Treatment adherence counsellors (*n* = 1)

^2^Counsellor at the clinic about risk of treatment failure (*n* = 1); Friend/peer/neighbor (*n* = 1); Peers and therapists at SU treatment (*n* = 1); Counsellor at the clinic in risk of treatment failure group (*n* = 1)

Three key themes were identified from the qualitative interviews: (1) patient-reported experiences of enacted SUD and HIV stigmas were common and impacted HIV care engagement; (2) patients and stakeholders considered a peer model highly acceptable for integration into HIV care to support engagement and address SUD stigma; (3) patients and stakeholders identified both individual-level and systems-level roles for peers, and key characteristics that peers would need to be successful in these roles. These themes are described below and illustrated with participant quotes.

### Patient and HCW experiences of HIV and SUD stigma were common and impacted HIV care engagement

Patients reported independent and intersecting experiences of stigma related to HIV and SUD that affected their motivation and engagement in HIV services. Most patient participants reported experiencing both HIV and SUD stigmas, including feeling stigmatized by structural factors related to HIV care delivery. For instance, patients described how the use of separate waiting rooms and clinic cards for patients with HIV compromised the confidentiality of their HIV status:

*“The way that our clinics are structured, that we are divided, like the waiting rooms…everyone can see that…this area specifically is for HIV positive people…people are very uncomfortable with that and they are worried about being seen by other people in the community...also our clinic cards are different…Those are the things that cause people to run away or not to attend clinic”* –Female, early 40s, patient

Additionally, several patient participants reported negative and punitive responses from HCWs about having contracted HIV and HIV adherence difficulties related to SUD, reflecting intersecting HIV and SUD stigmas. One patient participant shared:

*“He/she [the nurse] asked if I am taking my treatment and I told him/her that most of the time I forget because I drink, then he/she started shouting “What you are good at is drinking alcohol and opening your legs” and so I did not like how [they] spoke with me”* –Female, mid-30s, patient

Patients and stakeholders described experiences of intersecting HIV and SUD stigmas. Specifically, some patient participants described that when receiving punitive responses for non-adherence, HCWs often blamed substance use as the cause. In some cases, people may be refused HIV care for using substances. Some stakeholder participants confirmed that care refusal, although illegal, did sometimes occur:

*"I mean legally they can't refuse somebody treatment. But, you know, people are paternalistic and they would say, you know… ‘we are here to do the best for you but you need to take responsibility, so I'm not going to give you the treatment.’”* –Male, late 40s, stakeholder, HIV care

Several HCW participants used stigmatizing language when describing PWH and SUD, substantiating patient participants’ concerns about stigma from HCWs. For example, they described patients using substances as “untrustworthy,” that they might be dangerous or commit “crime.” Further, both patients and stakeholders were more critical of patients using non-cannabinoid drugs than cannabis and alcohol.

### A peer model was considered highly acceptable for integration into HIV care

Both patients and stakeholders perceived peers with lived experience of substance use as potentially helpful and destigmatizing members of an HIV care team, while noting potential implementation barriers. Although peers with SUD histories are not currently part of HIV care teams, some HCWs could see the potential benefits of this role based on their own interactions with people in SUD recovery. They described how informal interactions with people in SUD recovery shifted their attitudes towards PWH with SUD. One HCW shared:

*"It molded my way of thinking...I used to think recovery is just about leaving the drugs, but it's not the case… [interacting with a peer] altered my perception on the whole drug thing.”* –Male, early 30s, stakeholder, HIV care

Stakeholders who had worked in an SUD facility that incorporated program graduates as co-leaders of groups had positive perceptions of a peer model from these experiences. One HCW participant described the need for peers as recovery role models in their community:

*“People think a hero is only a sports star or musician or politician. But…those people, if they've recovered and they want to tell their stories, you can model them as community leaders.”* –Male, late 40s, stakeholder, HIV care

Further, one stakeholder acknowledged how integrating peers into the organizational structure could shift the overall culture to be more “welcoming” and destigmatizing.

In response to being asked how they felt about a peer model, patients described feeling “relieved” because of “shared understanding,” and that they would be able to “open [their] chest” and that “it’s all about asking from those who have walked the path.” Another patient contrasted their perceived comfort working with a peer with potential judgment from other providers without personal experience:

*“You feel comfortable when talking to someone who knows the shoes you are stepping in…you feel better, because sometimes you feel that, “How are they looking at me?” “Maybe they are pretending…” whereas when you know that this person has been through this road…other people would feel comfortable.”* –Female, early 30s, patient

A few patient participants described instances where HCWs had revealed lived experiences SUD and described how the sharing of this lived experience reduced their concerns about HCW stigma, making them feel more engaged in the treatment process:

“*I was relaxed. I was comfortable around the person. And I was free to talk about anything…I wasn't shy to ask questions about anything I wanted to ask about*.” –Female, mid-20s, patient

Some patient participants also indicated that they would be interested in being a peer themselves once in SUD recovery. They thought that this role would give them the opportunity to create meaning from their experiences and would allow them to serve as a role model for others struggling with similar issues:

*“I know that people want to hear mostly from a person who has experience. Someone who has also had struggles. I would love to help those people, tell them my struggles also.”* –Male, mid-30s, patient

Despite broad acceptability, participants did acknowledge possible barriers to implementation of the peer role. One potential barrier identified in implementing a peer model was HCW stigma towards SUD, such that HCWs may act in stigmatizing ways towards peers. For example, a few HCW participants expressed concern that someone with prior SUD would not be able to handle working with patients with active SUD, as it may threaten their personal recovery:

*“Maybe they come across something that affects them. I don't know, maybe a reason why they started using drugs in the first place... maybe there'd be triggers all around them that would maybe cause them to relapse.”* –Female, late 20s, stakeholder, HIV care

Other potential barriers included lack of private space in community settings, social and environmental factors that continue to contribute to relapse, and some HCWs perceiving that peers would be competing for their responsibilities (i.e., “take their place”), demonstrating the need for clarity for the peer role within the broader HIV care team. Further, it was noted that the peer role should be paid, yet one stakeholder noted that the peer may then experience separation from their community in a paid position, while also potentially not being considered fully part of the health care team.

### Individual and systems-level roles for peers and key characteristics required for role success

Both patients and stakeholders felt that peers could provide both HIV adherence support and SUD recovery support and serve as a bridge between patients and health care teams. Sufficient rapport between peers and patients and peers and health care teams was seen as critical to the success of these roles. More specifically, patient and stakeholder participants described roles for peers within a community-based HIV care team to better support patients to engage in HIV care and to support other HIV providers to engage more effectively with patients. They thought peers could support patients by providing reminders to take medication, attend HIV care appointments and by offering SUD recovery support. One patient participant shared:

*“I could gain information on his experiences, like what he used to do and how he recovered from that...maybe there are things that the person know that I do not know yet. And possibly his experiences, his past experiences he could share those with me and I could learn from them.”* –Male, early 40s, patient

In terms of systems-level supports, patient participants identified that peers could coach patients to advocate for themselves at HIV care visits, particularly in how to manage anticipated SUD stigma from HCWs or to help advocate for the patient with individual HIV providers. In this way, a peer would act as a bridge between patients and the HCW. One patient shared:

*“Even if you missed one day, you become scared of going because you know you are going to be shouted at, you will be asked a lot of questions, if there could be someone like that [peer] that you could talk to and someone who will at least understand you and that person could be the one who speaks directly with the nurses.”* –Female, mid-30s, patient

Stakeholders also identified a role for peers that involved helping providers better understand how to work with PWH with SUD. They provided suggestions for how peers could work alongside HCWs to enhance patient care:

*“The combination of both professional individuals as well as individuals in recovery…the registered individuals sort of come from a textbook environment…they come with the theoretical knowledge, but it's the individuals that have gone through recovery that understand the nuances and challenges of recovery…that really gives a more well-rounded package…it does help to have to have a recovering individual that can support the professional person…offer their perspective in sessions.”* –Male, late 30s, stakeholder, SUD care

Additionally, some stakeholders thought that peers could play a role in educating other HCWs in how to effectively communicate and engage with PWH with SUD in non-stigmatizing ways:

*“They are you know experienced...they just have to teach us how to approach maybe that patient or problem or whatever...teach us the basics and what to say and not what to say…Because sometimes the [HCW’s] attitude can also you know, change that person [impact the patient] and [lead to them] not hearing us.”* –Female, early 40s, stakeholder, HIV care

However, several stakeholders made suggestions that were likely unrealistic for the peer role, including getting the peer provider to address broader social determinants of health, social problems, and food insecurity.

Regarding characteristics necessary for peers to be successful in their role, patient and stakeholder participants almost uniformly agreed that the peer should have lived experience of SUD and SUD recovery, which they considered critical to their ability to support patients. Most participants reported that the peer should ideally also be living with HIV, though some stakeholder participants thought it would be sufficient if the peer had adequate information on living with HIV or had a close relationship with PWH. One provider said:

*“And if that person themselves is HIV positive, and adheres…they can be open about it…It just makes it more likely that someone would adhere…or be convinced to actually do it, because they've seen it, they've heard it, and the person is right there…evidence in front of them.”* –Male, late 30’s, stakeholder, SUD care

Apart from fluency in the same language, socio-demographic characteristics were seen as less important characteristics on which to match peers and patients. Language skills were seen as critical so that peers would be able to speak to and build rapport with patients, and some noted the importance of matching peers and patients on both race and language:

*“Sometimes when you want to talk about something, it's better to talk about it in your own language you know? If you are working in a Black community, let a Black person work there, somebody that they will understand...So if you are explaining something in your own language, then the person will understand you very well...you will also understand that person very well because it's in his or her own language. You must also consider race when it comes to hiring these people.” –*Female, late 30s, stakeholder, HIV care

Gender and age were mentioned by some participants as important for building rapport or for peer safety, mostly by stakeholders but also by some patients. Findings were however mixed; with other participants stating that age and gender should not matter when recruiting peers or matching peers and patients.

## Discussion

This study is among the first to explore the perceived acceptability of integrating peers into HIV care as a strategy for shifting SUD stigma at multiple levels and improving HIV care engagement among PWH who use substances. In keeping with previous studies [4–6], our findings confirm high levels of stigmatizing attitudes among HCWs towards PWH who use substances in SA, with stigma being portrayed in their language when describing patients who use substances. Our findings suggest that HCWs’ negative beliefs about PWH who use substances results in enacted stigma, with both HIV and SUD stigmas being experienced by patients and likely to be internalized. Given study findings that both anticipated and enacted stigma impact on PWH’s willingness to disclose substance use and their HIV care engagement, there is an urgent need for stigma reduction strategies that are feasible and scalable to implement.

Multi-level stigma reduction interventions that address both structural factors within health care services and HCW attitudes may be needed to shift HIV and SUD stigmas in this context [40–42]. Participants highlighted structural factors (e.g., separate HIV treatment clinics within primary care services) that inadvertently identified them as PWH, increased their anticipation of HIV stigma, and created potential for enacted stigma. Addressing this source of stigma would require a shift away from the vertical organization of HIV services towards horizontal integration of HIV care into general primary and community-based health services. In some parts of SA, an integrated chronic disease model that allows for HIV to be managed alongside other chronic diseases has been piloted [43, 44]. Should this model of care be implemented at scale, it may help reduce HIV stigma and possibly SUD stigma, and potentially allow for the incorporation of peers. This system reorganization will however take time to achieve; more immediate gains are likely through interventions that target HCW attitudes towards PWH who use substances.

Overall, our findings indicate that integrating peers into community-based HIV care may offer an acceptable solution to reduce HCW stigma. Although very few patients reported prior SUD treatment, all patients endorsed feeling comfortable talking to someone in SUD recovery and wanting them on their HIV care team. Stakeholders also reported high interest in receiving more training for working with patients with SUD. In qualitative interviews, HCWs and PWH perceived the peer role as acceptable, viewing peers as credible sources of information, experts in the SUD recovery process, and able to bring their lived experiences into interactions with patients and providers. Further, patients noted interest in becoming peer providers themselves. Very few barriers were noted regarding integrating peers into community-based HIV care. This may reflect a lack of familiarity with peer models, given this is not part of routine care in SA. However, opportunities patients and stakeholders had to interact with peers were seen as valuable to shift attitudes towards individuals with SUD, suggesting promise of the proposed model. Further, some stakeholders noted that peers and “professional” providers could partner to deliver evidence-based clinical care and provide complementary expertise and perspectives to enhance patients’ care experiences.

Although peers may be a potential strategy to shift HCWs’ attitudes towards people with SUD, HCWs’ stigma may be a barrier to the integration of peers. One stakeholder noted skepticism regarding whether individuals in recovery may be “triggered” as a peer and able to take on the challenges of the role. Most (75%) stakeholders had high levels of desired social distance for interacting with a patient with HIV and SUD when not in SUD recovery, yet the majority (90%) of stakeholders had low desired social distance when the patient had been in recovery for at least two years (a common threshold used for length of recovery in US peer recovery coach models). HCW attitudes and beliefs about recovery will be important to monitor as potential barriers to peer integration. Further, adequate supervision and ongoing support is needed for peers, particularly an approach that incorporates self-care and support to maintain peers’ own recovery amidst challenging work [24].

Findings suggest peers may play an important role in addressing multi-level stigmas, both potentially shifting internalizing stigma among patients and shifting HCW attitudes towards PWH with SUD. Some HCWs provided personal accounts of how their perceptions of people with SUD had shifted after engaging with peers. Stakeholders also noted that peers may be able to leverage their lived experience to build understanding among HCWs of the challenges patients face to enhance HCW empathy and facilitate patient-centered care. Further, and similar to studies in other contexts [24, 25], our findings suggest that peers may be able to support patients to overcome pragmatic concerns that impact care engagement, similar to patient navigators who facilitate linkage to HIV and tuberculosis care in SA, but have not addressed the intersection of HIV and SUD [45]. By incorporating their own lived experience with SUD [38], peers may also be able to help patients address SUD-related barriers to HIV care, navigate services, and advocate for their health needs.

Our findings point to the importance of clearly defining peer roles. Although patients and HCWs largely described roles for peers that aligned with typical expectations for peers, there were some examples of unrealistic expectations of the peer role (e.g., addressing broader social problems, food insecurity). Our team’s prior research in the US has demonstrated that a key barrier to integrating peers into health care teams includes lack of clarity for the peer role, which can contribute to tension within the health care team when not defined [24]. In the low-income, peri-urban areas where this study took place, there are multiple environmental factors and social determinants that are often barriers to SUD recovery that may not be feasibly addressed by the peer (e.g., poverty, unemployment, challenging social contexts and home environments, traumatic experiences). Developing clear, realistic roles for peers that capitalize on peers’ unique strengths should be prioritized. Yet, a strength of the peer role is also that they likely have shared some of these same experiences. Our team’s US-based work suggests that role clarification and communication between peers, health care teams, and organization leadership to clarify peer responsibilities is essential for successful integration of peers into new health care teams [24]. An advantage of peer recovery coach models is the flexibility to provide support outside the constraints of the clinic setting [24]; thus, future work is needed also to pilot different approaches for peer models in this context regarding location and treatment modalities, including implementation science research that evaluates strategies to promote integration of peers within existing health care teams [24]. Given how rapidly peer models for SUD have scaled in the past several years in the US [21], and the long history of peer models for mental health in the US, there may be valuable opportunities for bi-directional, mutual learning across high- and low-income contexts [46–48].

Findings must be considered in the context of study limitations. First, as this study first took a largely deductive approach guided by study aims to examine the experience of SUD stigma and perspectives on the peer role, we may have missed other themes outside of study aims that could have emerged from the data. Second, our patient sample was largely made up of Black women who used alcohol as opposed to other substances or demographic characteristics. Although this reflects the patient composition of HIV services in public clinics in SA, this may have limited our patient perspectives. Further, given the range of stakeholder perspectives we aimed to capture across HIV and SUD services, the sample size within each group was limited. However, our sample size determination was based on reaching theoretical saturation for our three primary study aims. Finally, given that peer models are limited in SA, some individuals may have had limited experiences to draw from in responding.

## Conclusions

Our findings suggest that peers may be acceptable to both patients and HCWs to promote more person-centered approaches for patients with SUD. Findings also highlight that for peer models to be feasible, HCW attitudes towards peers would need to be monitored and role expectations for peers clarified. As formal peer models for SUD do not widely exist in the SA health care system, identifying how this role would fit into existing care team structures and health system financing will be critical for sustainability. Evaluating the feasibility and acceptability of a peer role that involves structured training, supervision, and/or certification, as well as reimbursement, may help create a sustainable model for peer-delivered services in LMICs. This formative work points to the promise of further evaluating a peer model for reducing SUD stigma among patients and HCWs within community-based HIV care teams in SA.

## Supplementary Information

**Additional file 1.** Supplementary Material (SDS Vignettes).

## Data Availability

Data reported in this paper are available upon request.
