# Supplementary material for: “It’s all about asking from those who have walked the path”: Patient and stakeholder perspectives on how peers may shift substance use stigma in HIV care in South Africa
Source: Addict Sci Clin Pract. 2022 Sep 21;17:52. doi: 10.1186/s13722-022-00330-5 (PMC9490994; doi:10.1186/s13722-022-00330-5)
Supplement: Supplementary file 1 — Additional file 1. Supplementary Material (SDS Vignettes). [file 13722_2022_330_MOESM1_ESM.docx]

**Additional file 1**

| **Stakeholder Social Distance Scale (SDS) Vignette:** |
| --- |
| ***Directions:*** *Please read/listen to the following passage and imagine Andile. After reading/listening to the passage, please fill in the following questions about your responses to Andile.* |
| Andile started drinking and using dagga when he was still in school. He thought it was cool, but soon he started missing classes and it started to impact on his school work. He ended up dropping out of school and, after that, his whole life focused on hanging out with his friends and getting drunk and high. When he was 24, his girlfriend at the time became pregnant and he found out that both he and his girlfriend had HIV. Andile felt like his life was over and coped with these feelings by starting to use stronger drugs like tik. He thought he was in control of his drug use, but his appearance and behaviour changed. He got thinner and stopped taking care of himself. Although his brother managed to find him some part-time work in a construction job, he got fired because of inconsistent attendance on the job. He began stealing from his mother and sometimes from people in the community to pay for his drugs. His mother and brothers have tried to make him stop using drugs, but it often leads to arguments and conflict. Sometimes he gets into physical fights with his brothers. Andile's family does not know how to help him and are frustrated and aware that their neighbours are talking about what is going on in their home. Andile's mother is worried it is a matter of time before he gets arrested or killed. |

| **Patient (SDS) Vignette:** |
| --- |
| ***Directions:*** *Please read/listen to the following passage and imagine Andile. After reading/listening to the passage, please fill in the following questions about your responses to Andile.* |
| Andile started drinking and using dagga when he was still in school. He thought it was cool, but soon he started missing classes and it started to impact on his school work. He ended up dropping out of school and, after that, his whole life focused on hanging out with his friends and getting drunk and high. When he was 24, his girlfriend at the time became pregnant and he found out that both he and his girlfriend had HIV. Andile felt like his life was over and coped with these feelings by starting to use stronger drugs like tik. He thought he was in control of his drug use, but his appearance and behaviour changed. He got thinner and stopped taking care of himself. He stopped taking HIV treatment. His brother managed to find him some part-time construction work, and he started to get his life back on track. However, he needed additional support and his family found him a substance use treatment programme. With the structure of work and the treatment programme, he managed to stop using drugs. It has now been two years since he struggled with drug use. He also has been taking care of his health and regularly taking medication for HIV. His relationships with his family have improved. He would like to help others who are struggling with alcohol or other drug use. |

*NOTE: “Dagga” is a local word for marijuana. “Tik” is a local word for methamphetamine.*
